# Idiopathic Ventricular Fibrillation — Just How Much Idiopathic is it?

**DOI:** 10.31083/j.rcm2508306

**Published:** 2024-08-22

**Authors:** Samuel Lietava, Milan Sepsi, Tomas Novotny

**Affiliations:** ^1^Department of Internal Medicine and Cardiology, University Hospital Brno and Faculty of Medicine, Masaryk University, 625 00 Brno, Czech Republic

**Keywords:** idiopathic ventricular fibrillation, sudden cardiac death, arrhythmic syndrome, sudden cardiac death, long QT syndrome, Brugada syndrome, catecholaminergic polymorphic ventricular tachycardia

## Abstract

Idiopathic ventricular fibrillation is diagnosed in survivors of sudden cardiac death that has been caused by ventricular fibrillation without known structural or electrical abnormalities, even after extensive investigation. It is a common cause of sudden death in young adults. Although idiopathic ventricular fibrillation is a diagnosis of exclusion, in many cases only a partial investigation algorithm is performed. The aim of this review is to present a comprehensive diagnostic evaluation algorithm with a focus on diagnostic assessment of inherited arrhythmic syndromes and genetic background.

## 1. Introduction

Sudden death is a serious medical problem even in modern society. It is defined as a witnessed, non-traumatic and unexpected fatal event occurring within 1 hour of the onset of symptoms in an apparently healthy individual, or an unwitnessed death occurring within 24 hours after the individual was seen in good health [1]. If a cardiac cause is presumed as the reason for sudden death, or an autopsy has identified a cardiac anomaly as the probable cause of death (or in the absence of any obvious extra-cardiac causes at the post-mortem examination), it is referred to as sudden cardiac death (SCD) [2].

In Europe, SCD is the cause of death in 10–20% of all deaths, which equates to 300,000 individuals in Europe each year. The incidence of SCD differs according to age. It is very rare in childhood (1 per 100,000 person-years) [3, 4, 5], while in middle-aged individuals the incidence is 50 per 100,000 person-years [6, 7, 8]. The highest incidence is in the population over 80 years of age (200 per 100,000 person-years) [8]. The etiology of SCD depends on the age of the individual. In the elderly, ischemic heart disease and heart failure is the most common cause of SCD [9, 10]. On the other hand, congenital heart diseases, myocarditis and hereditary arrhythmic syndromes predominate among children and young people [3, 11, 12]. If structural heart disease, channelopathy, metabolic or toxicological etiology is excluded, then a diagnosis of idiopathic ventricular fibrillation (VF) is made.

Historically, ventricular tachycardia (VT) of variable duration followed by ventricular fibrillation as the dominant malignant rhythm responsible for cardiac arrest, was reported in most cases of SCD [13]. Only a minority of SCD victims had bradycardias or asystole [14]. However, more recent studies have shown shockable rhythm only in a minority of SCD victims [15, 16, 17].

## 2. Mechanisms of Ventricular Fibrillation

On an electrocardiogram (ECG), VF manifests as a disorganized and chaotic electric activity of the heart ventricles. The exact underlying mechanism of VF is not entirely clear. The widely accepted hypothesis declares four phases of VF (Table 1, Ref. [18]): initiation, transition, maintenance and evolution [19]. The initiation phase of VF typically starts by premature ventricular contractions (PVCs) or the degeneration of ventricular tachycardia. Rarely does VF start without PVC as the initial factor. The timing of PVCs falls into a vulnerable period resulting in R-on-T phenomena due to the short coupling interval. The origins of VF-related PVCs may be in any part of the heart ventricles. In the absence of structural heart disease, the majority of these PVCs arise from the right ventricle outflow tract (RVOT) [20], Purkinje system [21], papillary muscles [22, 23] and ventricular myocardium [24]. A recent theory proposes that four essential components are required to initiate re-entry: a local variation in excitability (for instance, steep gradients in repolarization time), a critical balance in the size between excitable and non-excitable regions, a trigger that emerges when some tissue is excitable while other tissue is not (such as an early premature complex), and the occurrence of this trigger from an excitable area (like a region with early repolarization) [25]. At the beginning, the PVC is a local phenomenon that may terminate spontaneously or degenerate into generalized VF. In the transition phase, PVCs generate wavefronts that propagate through a heterogeneous substrate and generate a wavebreak, multiple wavelets and slow conduction. In some cases, a functional re-entry is formed [26]. The VF re-entry circuits are not typical leading-edge loops but can be specific electrophysiological entities such as spiral waves and rotors. An inert core is localized in the centre and the activation propagates as a rotational wave. The evolution of the rotor with complex disorganized wavelets is the main point of the transition phase. Two mechanisms were proposed for VF maintenance. First, multiple unstable wavelets create a self-sustaining spiral wave re-entry. Another hypothesis suggests one “mother rotor” that is responsible for promoting new wavebreaks and driver formation [27]. However, both mechanisms can coexist and overlap each other [28, 29, 30]. Finally, rotors may establish at the scar borders, where anatomical or functional abnormalities are present.

**Table 1.** **Phases of VF with contribution factors**.

Phase	Initiation		Transition	Maintenance	Evolution
Contributing factors	∙ PVCs		∙ Alternans	∙ Focal sources	∙ Anchoring rotor to the scar
		- RVOT	∙ Wavebreaks	∙ Motor rotor	∙ Electrical remodelling
		- Purkinje	∙ APD heterogeneity	∙ Figure “eight” re-entry	
		- Papillary muscle	∙ Steep APD restitution		
	∙ VT		∙ CV restitution		

VF, ventricular fibrillation; PVCs, premature ventricular complexes; RVOT, right ventricle outflow tract; VT, ventricular tachycardia; APD, action potential duration; CV, conduction velocity. Modified from Anderson *et al*. [18].

## 3. Baseline Evaluation

The standard algorithm list for evaluating SCD survivors is presented in Table 2. This chain of examinations should be performed in every case of SCD.

**Table 2.** **Standard algorithm of examination in the case of SCD**.

Personal and family history
Physical examination
Blood tests, toxicology screening
Resting 12-lead ECG with right high leads
Coronary angiogram (CT coronary angiography in children and young adults)
Echocardiography
Cardiac magnetic resonance
Electrophysiology study
Exercise stress test
Pharmacological provocation test
Long-term ECG monitoring
Phenotype-guided genetic testing

SCD, sudden cardiac death; ECG, electrocardiogram; CT, computer tomography.

The clinical examination is standardized: blood tests, toxicology screening, 12-lead surface ECG, echocardiography and coronary angiography should be performed immediately. These examinations are usually sufficient to determine the etiology of the cardiac arrest. In some cases, however, it is necessary to search further. A post-VF patient without structural heart disease is indicated for an exercise stress test, pharmacological provocation test with sodium-channel blockers, long-term ECG monitoring and cardiac magnetic resonance (CMR). The diagnosis of idiopathic ventricular fibrillation (IVF) is a *diagnosis per exclusionem*, so only after all examinations mentioned above are performed can IVF be considered.

Real world data show that this diagnostic algorithm has been completed in only a limited number of IVF cases. Conte *et al*. [31] reported the registry of IVF survivors with a normal baseline 12-lead ECG where a complete workup (coronary angiography, CMR and sodium-channel blocker challenge) was performed in 46% of the patients, and an exercise stress test was performed in 80% of the group. Other authors have also published real world data of examinations of IVF survivors (Table 3, Ref. [31, 32, 33, 34, 35, 36, 37, 38, 39, 40]). The most recent and largest study published by Groeneveld *et al*. [32] shows similar numbers of examinations rates. These studies show the variability of the approach to patients after cardiac arrest and prove that a chain of evaluation has not been established world-wide.

**Table 3.** **Baseline evaluation in world-wide cohorts of IVF patients**.

Author	Numbers of patients	Cardiac coronary angiogram	Cardiac magnetic resonance	Exercise test	Sodium-channel blocker challenge
Krahn *et al*. (2009) [33]	63	100%	100%	100%	100%
Visser *et al*. (2016) [34]	107	100%	45%	100%	58%
Leinonen *et al*. (2018) [35]	76	NR	62%	75%	NR
Waldmann *et al*. (2018) [36]	49	100%	82%	8%	43%
Giudicessi *et al*. (2018) [37]	67	86%	73%	88%	27%
Conte *et al*. (2019) [31]	245	100%	65%	80%	64%
Frontera *et al*. (2019) [38]	54	44%	70%	83%	69%
Cunningham *et al*. (2020) [39]	46	11%	57%	41%	NR
Merghani *et al*. (2021) [40]	31	100%	77%	23%	81%
Groeneveld *et al*. (2023) [32]	423	96%	75%	70%	63%

NR, not reported; IVF, idiopathic ventricular fibrillation.

### 3.1 Personal and Family History

Anamnesis is the cornerstone of any diagnostic process. A detailed personal and clinical history from the patient him/herself and from family members is essential to provide insight into the potential etiology of SCD. A history of chest pain increases the probability of ischemic heart disease. Fever, hypothermia, dehydration can trigger life-threatening arrhythmia in genetic heart disease. For example, fever has been associated with malignant arrhythmias in 6% of patients with cardiac arrest and Brugada syndrome (BrS) [41]. An anamnesis of PVCs is associated with a higher risk of ventricular fibrillation [42]. Moreover, information that at first glance seems unimportant can be valuable. For example, an unexplained car accident in the past can be a sign of arrhythmic syncope. A family history of unexplained death, especially in young family members, is always a red flag and may provide an important clue to considering hereditary arrhythmic syndrome. Any banal unexplained accident with short unconsciousness by a family member should also be thoroughly investigated.

Medical history is very important clue too. Various drugs (also non-cardiological) may be proarrhythmic especially when used in combination. Many drugs are well-known for their potential for QT interval prolongation or provocation of a Brugada type 1 ECG pattern. There are websites with databases of potentially or definitive QT prolonging (http://www.QTdrugs.org/) or Brugada pattern provocation drugs (https://www.brugadadrugs.org/).

### 3.2 Physical Examination

Physical examination is the first step. This non-invasive examination can provide essential information and guide us to identify signs of syndromic and non-syndromic diseases that can be associated with SCD. Obesity and xanthomata may indicate coronary artery disease, while muscle weakness and atrophy may signalize lamin or desmin cardiomyopathies. Other physical signs include cardiac murmur in valve diseases, foetor ex ore in diabetic coma, and typical alcoholic or chemical foetor in an intoxicated patient. Body temperature must be measured repeatedly, and fever with rigor and shivering are signs of infection and can be a starter of VF associated with hereditary arrhythmic syndromes.

### 3.3 Blood Tests

Besides physical examination, paraclinical methods are also important for differential diagnostics. Standard blood tests targeted for levels of electrolytes, renal and liver function, high-sensitive troponin and other cardiac markers, and differential blood count are included in the standard protocol. Hypokalemia is prevalent in out-of-hospital cardiac arrest survivors due to the prolongation of the QT interval duration which canstart ventricular tachycardia [43, 44]. In any SCD survivor case, drug testing is required. Drug abuse is clearly associated with higher risk of arrhythmias, including VF [45, 46, 47]. In the absence of this information, the laboratory tests can be considered incomplete.

### 3.4. Resting Electrocardiography

A standard 12-lead ECG in patients after SCD may show various types of conduction abnormalities with dynamic changes in a short time, although a completely normal ECG curve is not rare. A detailed examination of a resting ECG with high right leads is recommended. PVCs may be present hours and days after ventricular fibrillation and resuscitation, the significance of which is commonly underestimated. Recording these PVCs on a 12-lead ECG is crucial for identifying the place of origin (left or right ventricle, outflow tract or Purkinje system). Non-RVOT or left ventricular PVCs are diagnostic criteria for arrhythmogenic cardiomyopathy (ACM) [48]. The PVC morphology may also be important for an ablation procedure in the future. Purkinje ectopias have a narrower QRS duration, especially from the left Purkinje system (duration QRS <120 ms) and a right bundle branch block morphology. A wider QRS duration (130–150 ms) with a left bundle branch block pattern is typical for Purkinje ectopias from the right Purkinje system.

An early repolarization ECG pattern (ERP) with typical J-point elevation on a 12-lead ECG is a common finding on ECG in the general population. In the ventricular arrhythmia-free population, the prevalence of ERP is generally reported from 5% [24] to 8.1% [49] and is more likely in young men and athletes [50, 51]. Early repolarization syndrome (ERS) is a different clinical entity [50, 52]. Haïssaguerre *et al*. [24] found ERS in up to 31% of IVF cases, compared to 5% in the control group. Mellor *et al*. [53] reported a significantly higher prevalence of ERP in SCD survivor relatives compared to the general population. In the absence of other clinical abnormalities, risk stratification and population screening are difficult to perform in patients with an ER pattern on resting ECG. High-risk ECG features have been proposed to increase the likelihood of ERS: prominent J-waves ≥2 mm, dynamic changes in J-point elevation (≥0.1 mV) and J-waves associated with a horizontal or descending ST-segment [50, 54, 55].

A specific group of IVF patients are individuals with VF induced by short-coupled PVCs. Generally, this group has not been excluded from IVF. It has been suggested as a new diagnostic entity—short-coupled VF (SCVF)—where the inducing factor of VF is a short-coupled PVC with a coupling interval <350 ms [56]. Steinberg *et al*. [57] retrospectively evaluated a cohort of patients in the CASPER registry to assess the proportion of SCVF. They reported a prevalence of 6.6% in the registry. Groeneveld *et al*. [58] in the Dutch IVF registry diagnosed SCVF in 14% of cases.

#### QT Interval Measurement

Correctly measuring the QT duration is extremely important. A study from Viskin *et al*. [59] showed that most physicians cannot find QT prolongation on pathological ECG. Like all ECG parameters, the QT interval also looks different in different leads. Historically, the QT interval was measured in lead II and the reference values are also determined for this lead [60]. In most children with long QT syndrome (LQTs), the longest QT interval was found in lead II as well [61]. In the situation where it is hard to recognize the start and end of the QT interval, lead I or V5 or V6 can be used, but the U-wave in the measurement of the QT interval must be avoided. A standard 12-lead ECG tracing at a paper speed of 25 mm/s and amplitude at 10 mm/mV is generally adequate for accurately measuring the QT interval duration in a healthy person, but for an accurate diagnostic algorithm, higher ECG paper speeds of 50 mm/sec may be used for identifying the low-amplitude waves such as U-waves. The QT interval should be determined as a mean value derived from at least 3–5 cardiac cycles and is measured from the beginning of the earliest onset of the QRS complex to the end of the T-wave [62]. In common practice, two measurement methods can be used - the threshold method and the tangential method. Currently, neither method is preferred. However, differences in the value of the measured QT interval are present [63]. The tangential method of measuring the QT [64] can be used for a difficult ECG, where the end of the T-wave is challenging to find, or the T- and U-waves are inseparable.

QT interval correction for heart rate is a disputable problem as old as ECG itself. Historically, in 1920 the two most commonly used correction formulas from Bazett [65] and Fridericia [66] were published. Because both formulas are logarithmic corrections, however, they are not accurate for slower heart rates under 60/min. Fridericia’s formula is considered to be more accurate than Bazett’s correction at faster heart rates above 100/min. These limits of both correction formulas must be mentioned in the cases of tachycardia or bradycardia.

In 2010, Viskin *et al*. [67] reported a simple test to detect corrected QT interval (QTc) prolongation in sinus tachycardia induced by orthostasis. In this study, the authors declared QTc prolongation after brisk standing in patients with LQTs compared to a control group. However, recent re-evaluation by Vink *et al*. [68] shows inconsistent findings. In light of these findings, the standing test is not superior to the standard ECG and exercise test for the diagnosis of LQTs. On the other hand, the standing test could be helpful for experts to stratify future arrhythmic risk.

Short QT syndrome (SQTs) is a rare condition characterized by short QT duration and premature atrial and ventricular fibrillation in the absence of structural heart disease [69]. According to current guidelines, SQTs are diagnosed in the presence of QT duration <320 ms alone or <360 ms and a history of aborted SCD or family history of SQTs or pathologic genetic findings (in genes *KCNH2*, *KCNQ1* and *SLC4A*) [2].

### 3.5 Cardiac Imaging Methods

#### 3.5.1 Coronary Angiography

In all SCD survivors, the anatomy of their coronary arteries must be well known to exclude coronary artery disease or congenital abnormalities. In the absence of pathological ST segment changes on the initial ECG, it is not necessary to perform emergency angiography. In young patients, computer tomography (CT) coronary angiography may be considered prior to invasive catheter coronary angiography.

#### 3.5.2 Echocardiography 

Echocardiographic examination is a first-line method to detect structural abnormalities of the heart. This low-cost, rapid, and radiation-free imaging method serves to visualize the heart wall motion and thickness, ejection fraction and valvular disease. However, it cannot be used to diagnose myocardial inflammation and local distribution of fibrosis.

With the development of the speckle tracking method, a subgroup of IVF patients with abnormal local and global deformation of the ventricles can be identified. Compared with healthy controls, IVF survivors showed more global deformation abnormalities as indicated by lower left ventricle (LV) global longitudinal strain and higher LV mechanical dispersion. Similarly, abnormal right ventricle deformation patterns have also been observed [70].

#### 3.5.3 Cardiac Magnetic Resonance

The development and increased availability of CMR in recent decades have provided us with an additional important tool for the diagnostic evaluation of SCD survivors. CMR findings can lead to a definitive diagnosis in cases of a normal echocardiogram [71]. In a study of 137 post-SCD patients, Neilan *et al*. [72] diagnosed or identified an arrhythmic substrate in up to 76% of all individuals after CMR was performed. Using T2 weighted sequences, specialists detected myocardial edema and fibrosis using late gadolinium enhancement. CMR enables the detection of myocardial inflammation, and confirmation of myocarditis completely changes the treatment and prognosis of the patient. There are, however, some genetic diseases which can mimic myocarditis. Desmoplakin cardiomyopathy can manifest as acute myocardial injury with a typical picture of inflammation on CMR. In this case, a genetic examination can provide the key to diagnosis [73]. To accurately measure right ventricle size and function, the role of CMR is crucial for diagnosing ACM, which is not only arrhythmic right ventricular cardiomyopathy (ARVC), as arrhythmogenic substrate can be expressed in the left ventricle or also in both ventricles. A group of authors from the Medical School of the University of Padua designed the Padua criteria for diagnosing ACM of the left ventricle by structural and functional abnormalities [48].

A relatively new entity in a subgroup of SCD survivors is mitral valve prolapse (MVP) and mitral valve disjunction (MVD). The incidence of MVP in an unselected population of SCD is generally documented below 1% [74, 75] but is reported from 4% [76] up to 11% in a subgroup of young patients with SCD [77, 78]. MVP is defined as a systolic displacement of one or both mitral leaflets ≥2 mm above the plane of the mitral annulus in the sagittal view of the mitral valve [79, 80, 81]. MVD is characterized by a systolic separation between the ventricular myocardium and the mitral annulus supporting the posterior mitral leaflet [82]. A patient with MVP/MVD may develop a wide variety of arrhythmias, from benign supraventricular premature beats to life-threatening ventricular arrhythmias and SCD. Mitral annulus disjunction and mitral valve prolapse are frequently overlooked and deserve extra attention in the extensive screening of patients with idiopathic ventricular fibrillation.

#### 3.5.4 Scintigraphy

The state of sympathetic innervation of the left and right ventricles can provide us with important information in the case of a high suspicion of ARVC and inconclusive CMR. Todica *et al*. [83] published a study with 123Iodine-Metaiodobenzylguanidine (123I-MIBG) single-photon emission computed tomography (SPECT)/CT to determine the quantity of right ventricle sympathetic innervation, which is lower in the case of ARVC compared to IVF. Another study by Siebermair *et al*. [84] demonstrated that impaired myocardial sympathetic innervation assessed by (123I-MIBG) SPECT is associated with cardiac events in a group of IVF patients.

### 3.6 Electrophysiology Study

Thanks to the development of better technology and research, the role of the electrophysiology study (EPS) in IVF cases is growing. While supraventricular tachycardias (SVT) are commonly benign, Wang *et al*. [85] reported a group of patients with SVT that deteriorated into VF. Other evidence of SCD caused by Wolf-Parkinson-White syndrome and other SVT has also been found [86]. The findings of these cases in a group of IVF patients are helping to rapidly change their prognosis. Another subgroup of patients with IVF shows PVCs triggering VF or localized structural alternations during EPS [87]. By modifying the triggering substrate VF events may be reduced and patients’ quality of life improved.

### 3.7 Exercise Stress Test

An exercise stress test should be a routine test in SCD survivors as it is safe, cheap, and widely available. In IVF cases this test is aimed at exercise-induced ventricular arrhythmias and assessment of the QT interval and T-wave morphology.

Catecholaminergic polymorphic ventricular tachycardia (CPVT) is still an underdiagnosed cause of cardiac arrest, especially in young individuals. While in resting conditions the ECG has no abnormalities, with increasing adrenergic stimulation polymorphic PVCs can occur. Bidirectional PVCs and/or polymorphic ventricular tachycardias are typical for a diagnosis of CPVT, and these arrhythmias can even degenerate into ventricular fibrillation. Data from studies confirm the importance of exercise stress testing [88]. Roston *et al*. [89] reported better results with a modified exercise test protocol with a sudden high workload at the beginning of testing to evaluate CPVT in a non-diagnostic standard protocol. Establishing the correct diagnosis has a great impact on further treatment, lifestyle recommendations and screening in the patient’s family.

An exercise stress test is highly valuable for detecting LQTs. Although QT interval prolongation on the baseline ECG is highly specific for LQTs, the QT interval often changes rapidly and may even be normal [90]. For a better diagnosis of LQTs, an exercise stress test is recommended [2]. It serves as an ideal method for physiologically altering autonomic tone, with good sensitivity and specificity for diagnostic LQTs [91]. Schwartz and Crotti [92] published a scoring system to establish the probability of LQTs. We currently use a modified Schwartz score (Table 4, Ref. [2]), where one part of the score represents exercise testing with QT interval prolongation of more than 480 ms at the 4th minute of recovery [2]. A score of more than 3 points means a definitive diagnosis of LQTs.

**Table 4.** **Modified LQTs scoring system**.

Findings			Points
ECG	QTc	≥480 ms	3.5
		460 ms–479 ms	2
		450 ms–459 ms	1
		≥ 480 ms in 4th minute of recovery from exercise stress test	1
	Torsades de pointes		2
	T-wave alternans		1
	Notched T-wave in 3 leads		1
	Low hearth rate for age		0.5
Clinical history	Syncope with stress		2
	Syncope without stress		1
Family history	Family member with definitive LQTs		1
	Unexplained SCD in first degree family member at age <30		0.5
Genetic testing	Definitive pathogenic mutation		3.5

ECG, electrocardiogram; QTc, corrected QT interval; LQTs, long QT syndrome; SCD, sudden cardiac death. Modified from 2022 ESC Guidelines [2].

### 3.8 Pharmacological Provocation Tests

BrS is diagnosed in patients without structural heart disease and a documented spontaneous Brugada type-1 ECG pattern or a history of syncope or SCD with a positive pharmacological provocation test [50, 93]. Brugada pattern ECG after the infusion of a sodium channel blocker does not automatically mean a diagnosis of BrS without other symptoms such as syncope or polymorphic ventricular tachycardia. This is a major change compared to the 2015 ESC Guidelines [1, 2] and leads us to be more specific with a diagnosis of BrS. The standard provocation test to uncover BrS is sodium channel blocker infusion with continuous 12-lead ECG monitoring modified with high right leads. Only a Brugada type-1 ECG should be considered as a positive result. Provocation tests are underused in clinical praxis. Conte *et al*. [31] reported that only 64% of patients underwent an ajmaline test. A repeated ajmaline challenge test may be considered to better detect BrS, especially in adults [94].

Coronary vasospasm is a rare cause of ventricular fibrillation. A provocation pharmacological test with ergonovine or acetylcholine can be used if coronary vasospasm is highly suspected as the etiology of SCD [95].

### 3.9 Prolonged ECG Monitoring

After an episode of SCD that is presented with VF, long-term ECG monitoring is also indicated. In general, men with frequent PVCs have an increased risk of SCD compared to controls. We look for PVCs, heart rate variability, bradycardias, ST segment changes and QT duration [96]. The duration of the QT interval may change according to physical activity, resting and emotional state, and variates in time. Long-term ECG recording may be more effective in monitoring the QT interval duration. Exercise-induced PVCs and ventricular tachycardia that do not manifest during a stress test in a medical centre may be presented during ambulatory ECG monitoring. Sitorus *et al*. [97] reported up to 33% recurrence of ventricular arrhythmias in individuals after SCD.

### 3.10 Genetic Background

For decades it has been well known that the occurrence of SCD has strong familial aggregation in the general population [98, 99]. Dekker *et al*. [100] showed that patients with a family history of SCD have a higher risk of VF during acute myocardial infarction (MI) compared to patients without a family history of SCD and acute myocardial infarction. All of these studies suggest a strong genetic component in SCD pathophysiology. Yet the precise mechanisms remain unclear. At the beginning of the “genetic era”, most clinicians expected simple answers, but with more and more information comes more and more questions. To date, many candidate genes have been reported for each inherited arrhythmic disease. Thus, genetic screening should have a high potential to identify individuals at risk for SCD. Nevertheless, a recent genome-wide association study (GWAS) exploring the genomics of SCD (the most extensive study of this type so far) failed to replicate an association of previously identified common genetic variants with SCD. Recently, the Clinical Genome Resource Consortium of the National Institute of Health has provided evidence-based curations of the clinical relevance of genes and gene-disease validity. Only genes with a classification of definitive or those with strong evidence supporting disease causation should be tested in patients with a clear specific phenotype [101].

#### Genetic Testing in IVF

According to current guidelines, IVF genetic testing related to channelopathy and cardiomyopathy may be considered with a mutation yield of 3–17% [2]. Most often, pathogenic variants related to LQTs, CPVT and BrS are identified [102, 103]. Interestingly, pathogenic variants in cardiomyopathy-related genes are also being found in a small proportion of idiopathic VF individuals despite the clinical exclusion of any structural abnormalities in these cases. This finding may suggest a latent structural underlying substrate and clinical phenotype could evolve later [104]. Nowadays, most IVF patients undergo genetic testing. However, the general detection of pathological gene variants is low. Verheul *et al*. [105] reviewed genetic results from a Dutch IVF registry. They reported findings of likely pathogenic/pathogenic (LP/P) variants in 15% of cases, including the risk haplotype *DPP6*. The most frequent LP/P variant found in cardiomyopathy-related genes was in* FLNC*, *MYL2*, *MYH7*, *PLN*, *TTN*, and *RBM20*. Change in diagnosis based on genetic testing results was present in 2% of cases. However, most gene variants were variants of uncertain significance (VUS) (30% of all results). This demonstrates the great uncertainty in the interpretation of genetic testing results in IVF patients. In common clinical practice, it is very difficult to establish a causal relationship between the IVF phenotype and the genetic findings of VUS.

LQTs is a condition with a prevalence of 1:2500 [106], which is borderline for rare diseases. In 2020, Adler *et al*. [107] revised all 17 genes previously associated with LQTs. Only the 3 oldest genes (*KCNQ1*, *KCNH2* and *SCN5A*) have been identified for phenotypically isolated LQTs causality. Another 4 genes (*CALM1*, *CALM2*, *CALM3*, *TRDN*) were found to have definitive evidence for causality in LQTs with atypical features. A list of LQTs-associated genes can be found in Table 5. Following this study, other genes are not recommended for genetic testing [101].

**Table 5.** **Genes associated with LQTs**.

Gene	Phenotype	ClinGen classification
*KCNQ1*	LQTs, JLNs	Definitive
*KCNH2*	LQTs	Definitive
*SCN5A*	LQTs	Definitive
*CALM1*	LQTs	Definitive
*CALM2*	LQTs	Definitive
*CALM3*	LQTs	Definitive
*CACNA1C*	Ts	Definitive in Ts
*KCNE1*	LQTs, JLNs, a-LQTs	Definitive in JLNs, Strong in a-LQTs
*KCNJ2*	ATs	Definitive in ATs
*TRDN*	Recessive LQTs	Strong
*KCNE2*	a-LQTs	Strong

LQTs, long QT syndrome; JLNs, Jervell Lange-Nielsen syndrome; ATs, Andersen-Tawil syndrome; a-LQTS, acquired long QT syndrome; Ts, Timothy syndrome.

The most common CPVT-causative gene is *RYR2 * [108, 109] with sporadic *de novo* variants. Other genes associated with the CPVT phenotype are *CALM 1-3 * [110], CASQ2, TRDN and *TECRL * [111, 112, 113, 114, 115] (Table 6). Similar to the LQTs’ “Schwartz score”, Giudicessi *et al*. [116] developed a clinical scoring system (without a genetic test result) for patients with a CPVT phenotype; for details see Table 7 (Ref. [116]).

**Table 6.** **Genes associated with CPVT**.

Gene	Phenotype	ClinGen classification
*RyR2*	CPVT – AD	Definitive
*TECRL*	CPVT – AR	Definitive
*TRDN*	CPVT – AR	Definitive
*CALM 1-3*	CPVT – AD	Strong
*CASQ2*	CPVT – AR	Strong
*CASQ2*	CPVT – AD	Moderate

CPVT, catecholaminergic polymorphic ventricular tachycardia; AD, autosomal dominant; AR, autosomal recessive.

**Table 7.** **Giudicessi scoring system for CPVT**.

Criteria		Points
Symptoms		
	Exercise-related SCD	2
	Exercise-related syncope or generalized seizures	1
Exercise stress test		
	Bi-directional ventricular tachycardia ≥100/min	4
	PVCs in bigeminy and bidirectional couples at HR ≥100/min	2
	PVCs at HR ≥100/min	1
Baseline QTc		
	≤420 ms	0.5
	421 ms–460 ms	0
	≥460 ms	–0.5
Genetic testing		
	Definitive pathogenic variant	4
	Likely pathogenic variant	2
	Variant of uncertain significance	0
	Negative findings	–1
ECG Holter examination		
	PVCs ≥2%	–1
Cardiac imaging		
	Structural or ischemic disease	–2
Age		
	≥50 years	–1
Family history		
	Definitive CVPT in first-degree relative	1.5
	Suspicious autopsy-negative SCD in first- or second-degree relative ≤45 years	1
	Unexplained autopsy-negative SCD in first- or second-degree relative ≤45 years	0.5

CPVT, catecholaminergic polymorphic ventricular tachycardia; SCD, sudden cardiac death; PVCs, premature ventricle complexes; HR, heart rate; QTc, corrected QT interval; ECG, electrocardiogram. Modified from Giudicessi *et al*. [116].

Concerning BrS, currently only the *SCN5A* gene (Table 8) has a definitive disease association. Approximately 20% of BrS cases are carriers of the* SCN5A* pathological variant [117]. The low number of this variant in the clinical presentation of BrS indicates a polygenic etiology of the disease. Cascade genetic screening is recommended in a family with a relative with BrS and an identified pathogenic or likely pathogenic *SCN5A* variant [101].

**Table 8.** **Genes associated with BrS**.

Gene	Phenotype	ClinGen classification
*SCN5A*	BrS	Definitive

BrS, Brugada syndrome.

### 3.11 Long-Term Follow-up

Even after the complete diagnostic algorithm and implantable cardioverter-defibrillator (ICD) implantation, it is advisable to periodically review the IVF diagnosis. Regular recording of ECG at each follow-up visit may reveal a latent form of inherited syndromes. When a phenotype of hereditary syndromes is suspected, also periodic stress testing should be considered. As shown by Merghani *et al*. [40] regular re-evaluation can increase the specific diagnosis from an initial 31% to 64%. Also, analysis of Dutch registry data shows a 9% increase in definitive diagnosis during follow-up [32]. Visser *et al*. [34] even report an increase in the number of definitive diagnoses during follow-up to 21%.

The specific diagnosis is also related to the prognosis of the patient. Survivors of sudden cardiac arrest (SCA) due to IVF have a high recurrence rate of arrhythmic events [31]. As shown in the Dutch registry analysis, patients with an alternative diagnosis have a higher chance of shocks compared to IVF patients. A correct diagnosis will therefore help to improve patient care with tailored treatment. However, Herman *et al*. [118] reported no significant difference in the number of ICD therapies between patients with IVF and alternative diagnoses in the CASPER registry.

## 4. Conclusions

IVF is still not very well understood, and the recommended diagnostic algorithm is often not systematically used. It seems that a significant portion of IVF in patients is only attributed to undiagnosed inherited electric syndromes. While modern advanced imaging methods can help us reveal more specific structural abnormalities, further research is needed in the field of echocardiography after IVF. Identifying the underlying etiology is essential for proper treatment and patient prognosis. With a correct diagnosis, we have the opportunity to reduce the recurrence of arrhythmias and to find family members at risk through family cascade screening. In the future, genome sequencing of true IVF survivors and their families, as well as computer models of cardiomyocytes, may shed more light on understanding the principles and substrate of IVF, which can lead to better healthcare for these patients. The result of this review should be an appeal for all clinicians to follow the described comprehensive algorithm for the examination of patients after SCD. It can be said that ICD implantation is not the end of the journey, but only the beginning.

## Abbreviations

ACM, arrhythmogenic cardiomyopathy; ARVC, arrhythmic right ventricular cardiomyopathy; BrS, Brugada syndrome; CMR, cardiac magnetic resonance; CPVT, catecholaminergic polymorphic ventricular tachycardia; ECG, electrocardiogram; EPS, electrophysiology study; ERP, early repolarization pattern; ERS, early repolarization syndrome; ICD, implantable cardioverter-defibrillator; IVF, idiopathic ventricular fibrillation; LP/P, likely pathogenic/pathogenic; LQTs, long QT syndrome; LV, left ventricle; MVD, mitral valve disjunction; MVP, mitral valve prolaps; PVCs, premature ventricular contractions; RVOT, right ventricle outflow tract; SCD, sudden cardiac death; SCVF, short-coupled ventricular fibrillation; SQTs, short QT syndrome; SVT, supraventricular tachycardia; VF, ventricular fibrillation; VT, ventricular tachycardia; VUS, variants of uncertain significance.
